# An Unusual Presentation of Spinal Giant Cell Glioblastoma in a 21-Year-Old Female

**DOI:** 10.1177/2324709619868255

**Published:** 2019-08-09

**Authors:** Benjamin J. Delgado, Leila Moosavi, Ericka Rangel, William Stull, Rahul Dev Polineni, Joseph Chen, Everardo Cobos

**Affiliations:** 1 Ross University, Miramar, FL, USA; 2 Kern Medical—University of California Los Angeles, Bakersfield, CA, USA

**Keywords:** giant cell, glioblastoma multiforme, GBM, primary spinal cord tumor, intramedullary

## Abstract

Primary spinal cord giant cell glioblastoma multiforme of the thoracic spinal cord is a rarely-diagnosed primary spinal cord tumor in comparison to neoplasms in intracranial locations. In this article, we highlight a young adult who was diagnosed with intramedullary giant cell glioblastoma, IDH wild-type, World Health Organization grade IV/IV of the thoracic spinal cord. This case report describes the treatment approach with a postsurgical combination of radiation therapy and temozolomide, which resulted in the patient to return to her baseline of health only to later develop neurological symptoms significant for a recurrence of malignancy. In a review of the literature of described cases of primary spinal cord glioblastoma multiforme, prognosis continues to be unfavorable as current treatment options of the aggressive malignancy remain absent of a cure.

## Introduction

The most common types of primary spinal cord tumors are astrocytomas and ependymomas.^1^ Ependymomas represent the most common spinal cord tumor among adults, while in the pediatric age group, astrocytoma of the spinal cord is more prevalent.^2^ Astrocytomas of the spinal cord alone occur with an incidence of 0.8 to 2.5 per 100 000 per year and are far less common than astrocytomas of the brain.^1^ Of the astrocytoma category of primary spinal cord tumors, glioblastoma multiforme (GBM) of the spinal cord has an extremely rare occurrence of 1.5% of diagnosed primary spinal cord tumors.^3^ Current therapies consist of radiation with chemotherapy, radiation without chemotherapy, surgery alone, surgery with adjunct therapy, and palliative therapy. As of April 2017, fewer than 200 cases of primary spinal cord glioblastomas have been documented in the literature and prognosis remains poor for the patients diagnosed with the disease.^4^ In a review of 190 documented cases of primary GBM of the spinal cord in the National Cancer Database, the median survival for patients is 11.2 months.^3^

## Case Presentation

A 21-year-old woman with no past medical history and of absent recent trauma presents with nonradiating lower back pain, bilateral lower extremity weakness with paresthesia, unsteady gait, and urine retention of 3-week duration. Her gait was normal during the first week of her symptoms. Later, she developed progressively worsening back pain and bilateral lower extremity weakness, greater in the right lower extremity, causing an unsteady gait with frequent ground-level falls. Neurological examination revealed preserved sensory function with intact cranial nerves I to XII, bilateral upper extremity strength 5/5, and the right lower extremity showed a 0 to 1/4 with a 2 to 3/5 strength in left lower extremity. There was no clonus or stiffness. Babinski sign was present bilaterally.

**Figure 1.**
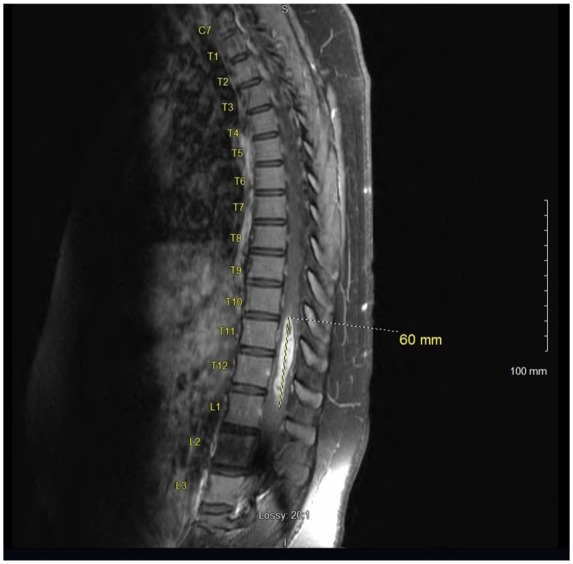
Preoperative sagittal MRI of the thoracic spine with contrast; significant for enhancing mass measuring 60 mm craniocaudal span.

**Figure 2.**
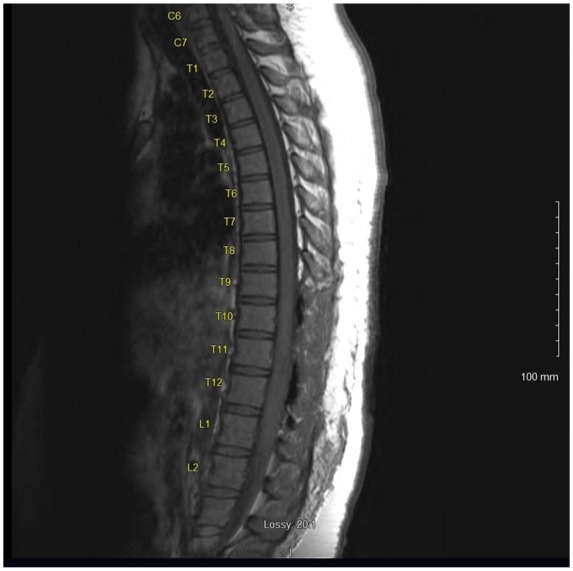
Preoperative sagittal MRI of the thoracic spine without contrast; significant for spinal cord mass at the level of T10-T12.

Magnetic resonance imaging (MRI) of the thoracic spine revealed a 60 mm enhancing intramedullary expansile mass of the lower thoracic spinal cord extending from T10-T11 down to the upper portion of L1 with a width of 10 mm × 9 mm transversely at the level of T12 (Figures 1 and 2). MRI of the brain, cervical spine, and lumbar spine were absent of any bleeding or mass. Additional imaging included a chest X-ray, which was normal.

**Figure 3.**
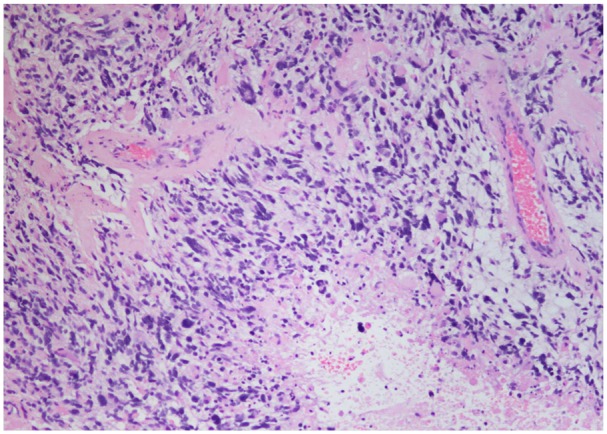
Neoplastic cells with vascular proliferation and focal necrosis on hematoxylin and eosin stains (100×).

**Figure 4.**
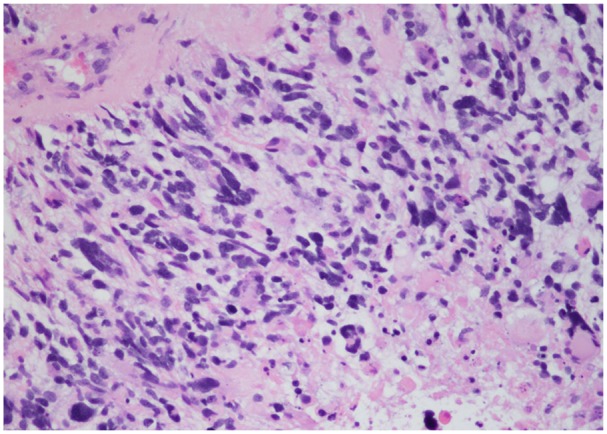
Neoplastic cells with focal necrosis on hematoxylin and eosin stains (200×).

**Figure 5.**
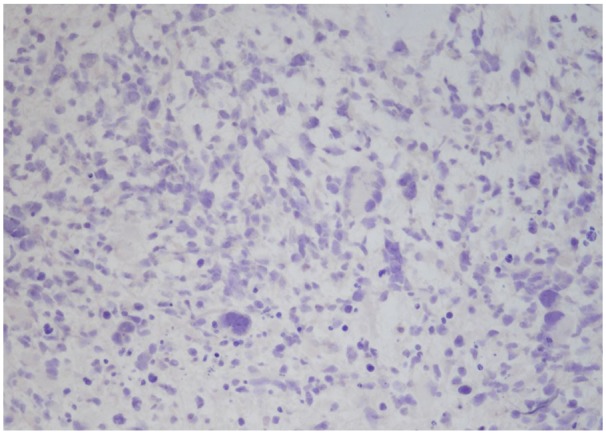
Negative immunohistochemical staining for isocitrate dehydrogenase-1 (200×).

**Figure 6.**
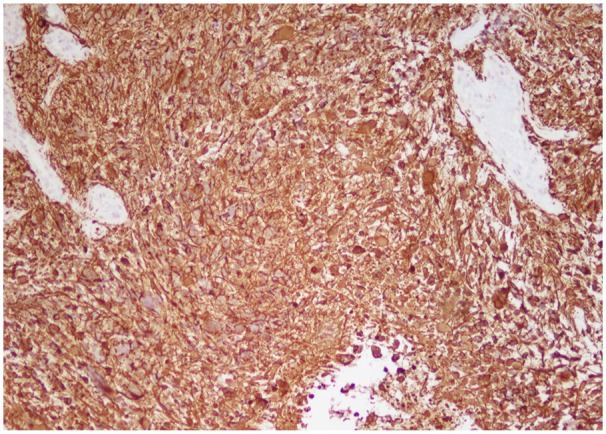
Positive immunohistochemical staining for glial fibrillary acidic protein (100×).

A laminectomy of T10-T12 and L1 with debulking of intramedullary mass was performed. Due to the nature of the intramedullary mass, complete tumor debulking could not be performed. Microscopic examination of the biopsied intramedullary mass was significant for neoplastic cells with vascular proliferation and focal necrosis on hematoxylin and eosin stains (Figures 3 and 4), immunohistochemical staining for isocitrate dehydrogenase-1 (IDH1) was negative (Figure 5), and immunohistochemical staining for glial fibrillary acidic protein was positive (Figure 6). These findings are significant for the diagnosis of giant cell glioblastoma, IDH wild-type, World Health Organization grade IV of the thoracic spinal cord.^5^ Further genetic testing for mutant-type IDH1 was not conducted on the tumor biopsies based on the negative IDH1 immunohistochemical staining.

**Figure 7.**
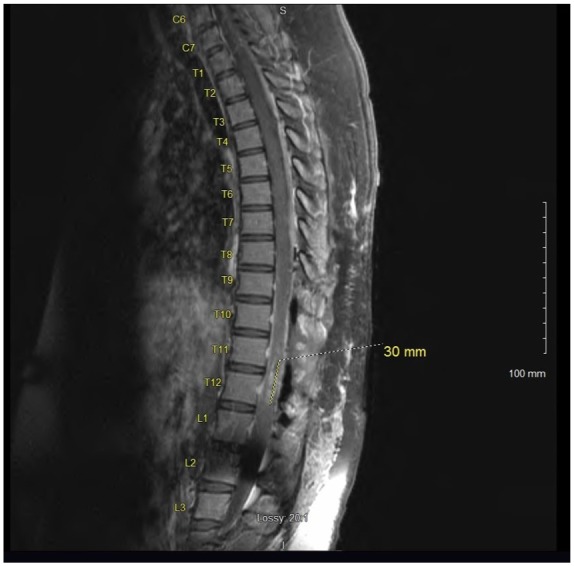
Postoperative MRI of the thoracic spine with contrast, postoperative day 1 of T9-T12 and L1 laminectomy with debulking of intramedullary spinal cord GBM. In this image, the residual tumor measures 30 mm in craniocaudal span.

**Figure 8.**
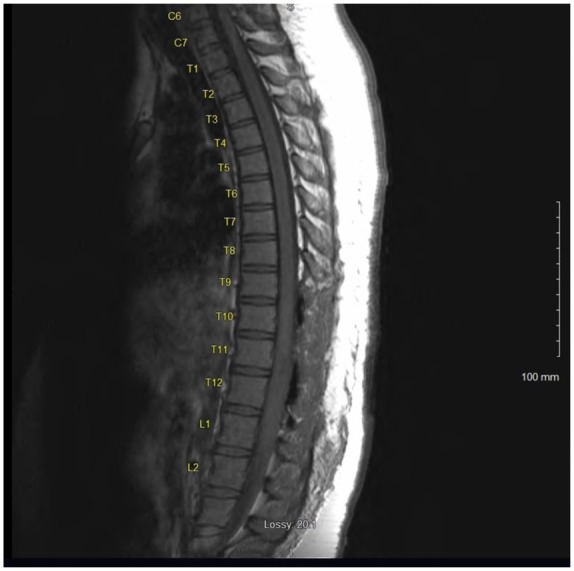
Postoperative MRI of the thoracic spine without contrast, postoperative day 1 of T9-T12 and L1 laminectomy with debulking of intramedullary spinal cord GBM.

On postoperative day 1, her urine retention resolved completely and she had significant improvement in her bilateral lower extremity weakness and paresthesia. Postoperative MRI of the thoracic spine with contrast was repeated on postoperative day 1, which showed a laminectomy of T9-T10 and L1 with a significant residual enhancement of intramedullary GBM at the level of T12. Compared with previous imaging, there was a reduction of the primary GBM of 60 mm reduced to 30 mm in craniocaudal span at the level of T12 with no abnormal spinal fluid collection (Figures 7 and 8).

Further management of this patient consisted of adjunct chemoradiation of 1440 Gy with temozolomide 75 mg/m^2^ (125 mg daily) during the course of radiation therapy, followed by maintenance temozolomide 150 mg/m^2^ (250 mg daily) for days 1 to 5 every month planned for a total of 6 months. The results of the tumor debulking surgery followed by adjunct therapy for giant cell GBM of the spinal cord resulted in resolution in back pain and lower extremity symptoms. The patient was able to return to her baseline level of activity about 2 months after the initial presentation.

She remained asymptomatic of any neurological deficits for about 4 months after surgery, only to later develop a recurrence of left-sided lower back pain with right lower extremity pain and paresthesia.

## Discussion and Literature Review

Spinal cord tumors can occur within or adjacent to the spinal cord. They are located intra-axially and can be either primary or metastatic. Primary spinal cord tumors account for 2% to 4% of all primary central nervous system tumors, with one third of them located in the intramedullary compartment.^6,7^ Ependymoma is the most common intramedullary spinal cord tumor in adults, with the peak age at the time of presentation reported to be between 30 and 40 years.^8^ Among the pediatric age population of patients 21-year-old and younger, intramedullary spinal cord tumors make up 1% to 10% of diagnosed central nervous system neoplasms, with astrocytomas being the most common tumor subtype of 40% to 60% of diagnosed intramedullary spinal cord tumors.^8^ The astrocytoma neoplasm of GBM is far less common among spinal cord tumors and represents only 1.4% of intraspinal tumors.^9^

Our patient was diagnosed with high-grade GBM, World Health Organization grade IV at the age of 21 years after the initial-onset subacute back pain that progressed to the development of neurological deficits of urine retention and lower extremity motor weakness. The presentation of atraumatic back pain in a young adult is an uncommon presentation, with a rarer presentation of intramedullary giant cell GBM. GBM is an aggressive, high-grade malignancy of glial cell origin.^9^

Shibahara et al described that the incidence of spinal metastasis is relatively high in primary intracranial glioblastoma, and these patients will benefit from spinal screening to detect any early asymptomatic spinal metastasis.^10^ Unlike the spinal metastasis from an intracranial primary, the intracranial metastasis from a spinal source is not that common. Reviewing the literature reveals just a few reported cases of primary GBM spinal cord tumors with the characteristic of metastasis to the brain.^10,11^ Neuroimaging of the brain and full spinal cord by MRI with T1- and T2-weighted sequences and/or with perfusion imaging can help identify if the neoplasm is a primary lesion or a secondary metastasis.

Reviewing the National Cancer Database of 103 496 patients diagnosed with primary brain GBM versus 190 patients diagnosed with primary spinal cord GBM between the years of 2004 and 2014 showed that primary spinal cord GBM had a greater mean survival of 11.2 months compared with cranial GBM median survival age of 9.2 months.^3^ Among these 190 spinal GBM, 18 to 65 years age group reported having a longer survival time of 13.2 months, compared with younger than 18 years old and older than 65 years old groups with 11.9 months and 3.9 months, respectively. No clear explanation was identified in this cohort-based study.^3^

Closer examination of 95 patients with spinal cord GBM with clear documented treatment plan registered in the national cancer database between the years 2004 and 2014 revealed that the overall survival time was not statistically different among the 5 various treatments options the patients received of either radiation with chemotherapy, radiation without chemotherapy, surgery alone, surgery with adjunct therapy, and palliative therapy.^3^ The chemotherapy type and details of radiotherapy were not specified in this study. Despite this limitation, this study did reveal that gross total resection or subtotal resection of the spinal cord led to more morbidity and GBMs are introduced as highly infiltrative malignancies in nature.^3^ Moreover, they found that overall survival in these malignant lesions was found to be worse in patients younger than 18 years old compared with patients between 18 and 65 years -old.^3^ The possibility of reoccurrence in such an aggressive malignancy has never been investigated.

Because the occurrence of primary spinal cord GBM neoplasm is rare, there has been limited reported cases of the management in the literature, and to the best of our knowledge, no clinical trials for the management of primary GBM spinal cord neoplasms have been conducted. Historically, spinal cord GBM has been treated as intracranial GBM with combination of radiation therapy and temozolomide. Current management of these patients include combination therapy of surgery, chemotherapy, and radiation therapy. The multimodal treatment of surgery, chemotherapy, and radiation therapy approach remains absent of a cure of spinal cord GBM but has demonstrated to improve the overall quality of life in these patients.^3,12,13^

The alkylating agent, temozolomide, and monoclonal antibody against VEGF, bevacizumab, have been described in the literature to increase the median survival time for patients with intramedullary GBM.^12-15^ When the use of temozolomide was compared with non-temozolomide patients with primary spinal cord GBM, the median survival time was 16 months and 10 months, respectively.^14^ When the antiangiogenic effect of bevacizumab as salvage therapy was examined among 6 patients with recurrent primary spinal cord GBM, median overall survival rate was 22.8 months.^15^ This new recombinant humanized monoclonal antibody, bevacizumab, has been reported to have a tolerable toxicity panel and has been well-tolerated among the patients with recurrent spinal cord glioblastoma.^6^ The use of combination of temozolomide with bevacizumab in patients with primary spinal cord GBM has not been conducted.^16^ Current guidelines suggest management of intramedullary spinal cord GBM with the chemotherapy drug temozolomide, as part of aggressive multimodal treatment.^13^

Grade IV GBM is a highly malignant and vascularized tumor. Due to its aggressive nature, the patient was unable to successfully undergo complete surgical debulking of the tumor. Nevertheless, when the patient was reevaluated at her 2-month postoperative follow-up visit, her neurological deficits had resolved. The patient essentially returned to her baseline of health, began to ambulate independently, returned to work, and completed a semester of college classes. Despite excellent medical chemotherapy radiation adherence, the patient later developed reoccurring neurological deficits of the right lower extremity including numbness as well as right-sided lower back pain. This prompted investigation for possible reoccurrence of malignancy.

## Conclusion

Primary GBM of the spinal cord remains a rare diagnosis of spinal cord tumors. No different than well-documented primary cranial GBM, treatment options remain limited to neurosurgery and adjunct radiation with chemotherapy. It is questionable that advances in the treatment of primary cranial GBM can lead to more favorable outcomes in patients with primary spinal GBM; therefore, further exploration and development of therapies for GBM should be continued to be studied.
